# Phase II trial evaluating the clinical efficacy of cefixime for treatment of active syphilis in non-pregnant women in Brazil (CeBra)

**DOI:** 10.1186/s12879-020-04980-1

**Published:** 2020-06-10

**Authors:** Melanie M. Taylor, Edna Oliveira Kara, Maria Alix Leite Araujo, Mariangela Freitas Silveira, Angelica Espinosa Miranda, Ivo Castelo Branco Coelho, Maria Luiza Bazzo, Gerson Fernando Mendes Pereira, Silvana Pereira Giozza, Ximena Pamela Díaz Bermudez, Maeve B. Mello, Ndema Habib, My Huong Nguyen, Soe Soe Thwin, Nathalie Broutet

**Affiliations:** 1 Department of Sexual and Reproductive Health and Research, World Health Organization, Geneva, Switzerland; 2 U.S. Centers for Disease Control and Prevention, Division of STD Prevention, Atlanta, GA USA; 3 University of Fortaleza (UNIFOR), Fortaleza, Brazil; 4 Post-Graduate Program in Epidemiology, Federal University of Pelotas, Pelotas, Brazil; 5 Federal University of Espírito Santo, Vitória, Brazil; 6 Federal University of Ceará, Ambulatório de IST do Hospital Universitário da Universidade Federal do Ceará, Fortaleza, Brazil; 7 Federal University of Santa Catarina, Florianópolis, Brazil; 8 Brazil Ministry of Health, Secretariat for Health Surveillance, Department of Chronic Conditions and Sexually Transmitted Infections, Brasília, Brazil; 9 University of Brasília, Brasília, Brazil; 10 Department of Communicable Diseases and Environmental Determinants of Health, Pan American Health Organization/World Health Organization, Washington, Washington, DC, USA

**Keywords:** Syphilis, cefixime, Benzathine penicillin, Congenital syphilis, Sexually transmitted infections, Alternative treatment

## Abstract

**Background:**

Syphilis is a sexually and vertically transmitted infection caused by the bacteria *Treponema pallidum* for which there are few proven alternatives to penicillin for treatment. For pregnant women infected with syphilis, penicillin is the only WHO-recommended treatment that will treat the mother and cross the placenta to treat the unborn infant and prevent congenital syphilis. Recent shortages, national level stockouts as well as other barriers to penicillin use call for the urgent identification of alternative therapies to treat pregnant women infected with syphilis.

**Methods:**

This prospective, randomized, non-comparative trial will enroll non-pregnant women aged 18 years and older with active syphilis, defined as a positive rapid treponemal and a positive non-treponemal RPR test with titer ≥1:16. Women will be a, domized in a 2:1 ratio to receive the oral third generation cephalosporin cefixime at a dose of 400 mg two times per day for 10 days (*n* = 140) or benzathine penicillin G 2.4 million units intramuscularly based on the stage of syphilis infection (*n* = 70). RPR titers will be collected at enrolment, and at three, six, and nine months following treatment. Participants experiencing a 4-fold (2 titer) decline by 6 months will be considered as having an adequate or curative treatment response.

**Discussion:**

Demonstration of efficacy of cefixime in the treatment of active syphilis in this Phase 2 trial among non-pregnant women will inform a proposed randomized controlled trial to evaluate cefixime as an alternative treatment for pregnant women with active syphilis to evaluate prevention of congenital syphilis.

**Trial registration:**

**Trial identifier:** www.Clinicaltrials.gov, NCT03752112. Registration Date: November 22, 2018.

## Background

### Epidemiology of syphilis

Syphilis, caused by the bacterial spirochete *Treponema pallidum,* is a global health problem. In 2016, the World Health Organization (WHO) estimated 6.3 million new cases of syphilis worldwide, of which 1 million were pregnant women. An estimated 660,000 cases of congenital syphilis occurred in 2016 including 350,000 adverse birth outcomes of which, over 200,000 presented as stillbirths or neonatal deaths [1]. Among adults with undiagnosed syphilis, progression to organ and neurologic system manifestations (neurosyphilis, stroke) occur at a faster pace among those co-infected with HIV [2]. The highest burden of adult and infant infections is within the African region [1, 3]. Early identification of syphilis and prompt treatment can eliminate the risk of serious clinical manifestations and reduce transmission to partners and newborns.

### Transmission and clinical stages of syphilis infection

The primary route for transmission of syphilis is through sexual contact. Primary stage infection is heralded by a painless “chancre” at the site of exposure. Without identification and treatment infected persons progress to the secondary stages whereby dissemination of the bacteria is manifested in skin lesions that can occur in the mouth, the palms and soles, the torso, legs, face and genital region (condyloma lata). Failure to diagnose and treat the secondary stage allows progression to early (less than 2 years from time of infection) and late latent stages (greater than 2 years since infection) whereby continued dissemination, neurologic invasion and organ involvement can occur. The primary, secondary, and early latent stages are those for which risk of transmission to both sexual partners and infants during pregnancy are the highest [2].

Syphilis can be transmitted from mother to fetus during pregnancy or at birth, resulting in congenital syphilis. Untreated syphilis during pregnancy can lead to fetal loss or stillbirth or, in a live-born infant, neonatal death, prematurity, low birth weight or syphilis infection in the infant. In syphilis-infected pregnant women, adverse birth outcomes are common and have been shown to be 4.5 times higher in those with untreated syphilis than those without syphilis [4]. Congenital syphilis can be prevented by screening early in pregnancy, treating seropositive pregnant women, and preventing re-infection [5].

### Current treatment recommendations

Penicillin is the primary recommendation for treatment of all stages of syphilis by both the WHO and the U.S. Centers for Disease Control and Prevention [5–7]. The efficacy of penicillin for the treatment of syphilis, however, has primarily been established through clinical experience prior to the time when the value of randomized controlled clinical trials was recognized. Thus, almost all the recommendations for the treatment of adult syphilis and congenital syphilis are based on expert opinion reinforced by case series and more than 50 years of clinical experience [7].

Based on clinical evidence, doxycycline is recommended as an alternative to penicillin for non-pregnant patients that are allergic to or otherwise cannot tolerate penicillin injections [6]. Doxycycline, however, is contraindicated in pregnant women. WHO recommends erythromycin as an alternative treatment for pregnant women with syphilis who cannot receive benzathine penicillin G (BPG) but recognizes that this macrolide does not cross the placenta in amounts sufficient to treat the foetus. Thus, WHO recommends that infants born to mothers with syphilis that received treatment with erythromycin be treated for congenital syphilis with penicillin [5].

## Background and rationale

### BPG shortages and the need for alternative therapies for pregnant women with syphilis

During 2014–2016, Brazil experienced a nationwide shortage of BPG. This coincided with global shortages of BPG reported by more than 39 countries [8, 9]. During this period some pregnant women with syphilis went untreated, resulting in adverse birth outcomes due to congenital syphilis. Pregnant women in Brazil and other countries experiencing BPG shortages were treated with alternative medications for which efficacy data for the prevention of congenital syphilis are not available [8].

### Alternative candidates for evaluation of efficacy in treatment of syphilis

#### Ceftriaxone

A recent meta-analysis of seven randomized controlled trials and 281 patients with syphilis demonstrated no significant difference in the 6-month or 12-month response rate in patients treated with ceftriaxone, an intravenous/intramuscular (IV/IM) third generation cephalosporin similar in antimicrobial spectrum to cefixime, compared with those treated with penicillin. These analyses demonstrated no significant difference in relapse rate, serofast rate, or treatment failure rate in patients treated with ceftriaxone compared with those treated with penicillin [10]. A multi-centre trial comparing penicillin with ceftriaxone found similar results [11]. There were no pregnant women in these trials. The minimum inhibitory concentration (MIC) and minimum bactericidal concentration (MBC) of ceftriaxone in treatment of *T. pallidum* infection are low, 0.0007 and 0.002 respectively and are similar to those of penicillin [12].

Third generation cephalosporins such as ceftriaxone and cefixime (pregnancy category B) are known to cross the placenta but treatment doses, efficacy, and duration of therapy in pregnant women with syphilis have not been evaluated in prospective trials [13–15]. Other oral or IV penicillin derivatives such as amoxicillin and ampicillin (pregnancy category B) have been used with success in treatment of syphilis [16–18]. However, only a few case reports and one retrospective case review are available describing use of these medications in pregnant women [19–22].

#### Amoxicillin

One retrospective analysis and a few case reports are available describing use of amoxicillin for the treatment of pregnant women with syphilis [19–22]. The retrospective analysis of infant cases born to women with active syphilis (treponemal test positive, and RPR titer ≥1:8) in Japan showed that congenital syphilis was prevented among women with early stage (primary, secondary or early latent stages) syphilis after receiving amoxicillin [21]. A recent report describes two pregnant women successfully treated and congenital syphilis prevented with amoxicillin and probenecid in the first case, and ceftriaxone in the second case [22]. No clinical trials have been reported comparing these medications to the recommended treatment of BPG in pregnant women.

Treponemicidal concentrations of amoxicillin in cerebrospinal fluid have been reached with oral administration of amoxicillin among patients with neurosyphilis [23, 24]. Amoxicillin effectiveness has been established at a ratio of 23:1 compared to sodium penicillin G. Thus, a level of 0.42 μg of amoxicillin should be reached for treponemicidal activity using the WHO-recommended level of 0.018 μg/ml of penicillin [25]. Amoxicillin renal excretion is higher and the half-life is shorter during pregnancy as compared to postpartum, particularly in the second and third trimesters. This supports the need for more frequent dosing to meet the minimum inhibitory concentrations (MICs) for treponemicidal activity. Amoxicillin is well tolerated, and higher peak concentrations are reasonably safe to ensure adequate trough and steady state concentrations [26]. The effective concentrations of penicillin in vivo have been evaluated for *T. pallidum*, in mouse and rabbit models demonstrating treponemicidal activity is improved with longer duration of penicillin exposure [27].

### Selection of cefixime for this phase II trial

Cefixime is an orally administered third-generation cephalosporin with spectrum of activity similar to that of ceftriaxone that may be administered with or without food [28]. Unpublished results in rabbits demonstrate that cefixime is active against *T. pallidum* during early infection (Sheila Lukehart, personal communication, Seattle, WA USA). Protein binding is limited and bioavailability with oral dosing is about 45%. Cefixime is moderately distributed into extracellular water/tissue pools. A majority of systemically available cefixime is eliminated by biliary excretion [29]. Adverse event profiles are favourable with cefixime in non-pregnant and pregnant patients [30]. Cefixime has been evaluated in pregnant patients for treatment of urinary tract infections [31, 32]. Cefixime is detectable in amniotic fluid at a level of 0.85 ± 0.42 μg/mL [15], levels approximately 2-fold higher than the MIC for *Treponema pallidum* [12]. Cefixime has a high amniotic fluid passage rate and therefore can be considered for use as a therapeutic agent in infectious conditions in which membranes and the placenta are involved [15]. The drug has a well-known pharmacokinetic profile with common use for treatment of *Neisseria gonorrhoea*. Typical blood levels after a single dose of cefixime 400 mg by mouth are 4.84 μg/ml maximum at four hours and above 1.0 μg/ml at 12 hours [30]. The antibiotic, cefixime, used in this study has received U.S. Food and Drug Administration (FDA) marketing approval for use in the U.S. for at more than one clinical indication by the FDA, and has been in use for several years.

*This Phase II trial will be conducted only in non-pregnant women to determine efficacy of cefixime. Consideration for a future randomized controlled trial among pregnant women will depend on the results of this study.*

### Selection of study sites in Brazil

Brazil is committed to the identification of alternative treatment options for pregnant women with syphilis to avoid adverse birth outcomes. In addition, this country is experiencing increases in syphilis among general populations, including pregnant women, further straining BPG stock and procurement. Three cities in Brazil will be sites of participant enrolment: Fortaleza (Northeast), Espirito Santo (Southeast), and Pelotas (South). Each of these cities has experienced an increase in syphilis among pregnant and non-pregnant women over preceding years and represent committed local sites for this study.

### Rationale and public health importance of identifying alternative treatments for syphilis

Prompt treatment of people diagnosed with syphilis is paramount to reduce sexual and vertical transmission. Treatment with BPG has been hampered by periodic and prolonged shortages in numerous countries [8]. These shortages signify an urgent need to ensure stable supplies of BPG for treatment of maternal and congenital syphilis while evaluating alternative treatment options. Identification of alternative treatments for syphilis has been identified as a research need and priority by WHO [3, 4].

The public health impact of identifying alternative treatment options for those with syphilis would be considerable and could contribute to reducing global burden and to support the effort to eliminate mother-to-child transmission of syphilis (congenital syphilis). Identifying treatment alternatives for syphilis could ensure that people are appropriately treated during periods or settings of BPG stock out, penicillin allergy, or other intolerance to penicillin injection. This study may also identify an oral regimen for settings in which injections are not feasible.

### Ethics approval and consent for participation statement

This protocol was approved by (1) the World Health Organization Ethics Review Committee (WHOERC), (2) Comissão Nacional de Ética em Pesquisa (CONEP), (3) Universidade de Fortaleza (UNIFOR), (4) Centro de Ciências da Saúde da Universidade Federal do Espirito Santo (UFES), (5) Faculdade de Medicina da Universidade Federal de Pelotas (UFPel), (6) Hospital Universitário Walter Candido da Universidade Federal do Ceará (UFC), and (7) the U.S. Centers for Disease Control and Prevention, National Center for HIV, Hepatitis, Sexually Transmitted Diseases, and Tuberculosis Prevention (CDC). Important protocol modifications (eg, changes to eligibility criteria, outcomes, analyses) will be communicated to ethics review committees, study investigators, trial participants, and trial registries.

All eligible participants must sign a written consent prior to enrolment in this study.

### Intervention

This study will evaluate an alternative treatment option as an intervention to manage syphilis.

### Study hypotheses

The antibiotic, cefixime, for use in non-pregnant women with acute syphilis will be efficacious and safe.

### Primary objective

The primary objective of the study is to demonstrate the efficacy, as measured by a 4-fold (or more) decrease in rapid plasma reagin (RPR) titer from baseline to 6 months after treatment, with cefixime 400 mg taken orally two times a day for 10 consecutive days.

### Secondary objective

The secondary objective of the study is to determine the safety, as measured by the percent of treated patients experiencing mild, moderate, severe, or life-threatening adverse events associated with the study product.

## Methods/design

This is a randomized, non-comparative, open label study assessing the efficacy and safety of cefixime 400 mg taken orally two times a day for 10 consecutive days in non-pregnant women with active syphilis infection.

### Study population and enrolment sites

Numerous regions of Brazil have experienced increases in syphilis diagnoses during the previous five years [33]. The study sites were chosen from three cities in three separate regions (states) of Brazil based on: (1) increases in syphilis case reports; (2) access to clinics where patients are screened and treated for syphilis; (3) willingness of clinic sites and staff to participate in this research; and (4) the availability of researchers with experience in studying syphilis to serve as co-investigators.

The study population will include non-pregnant women (ages 18 and over) diagnosed with early syphilis (RPR titers ≥1:16) recruited from six clinics in three cities from three states in Brazil (Table 1). These include: (1) Three clinics in the city of Fortaleza (state of Ceara), coordinated by the UNIFOR: (a) Centro de Saúde Carlos Ribeiro, (b) Centro de Saúde Meireles, and (c) Ambulatório de ITS - UFC; (2) Two university obstetrics and gynecology (ObGyn) clinics in the city of Pelotas (state of Rio Grande do Sul): (a) Ambulatório de Ginecologia e Obstetrícia - Faculdade de Medicina - UFPel and (b) Ambulatório de Ginecologia e Obstetrícia - Campus da Saúde - Universidade Católica de Pelotas; and (3) One clinic in Vitoria (state of Espirito Santo): (1) Ambulatorio de Ginecologia e Obstetrícia, Hospital Universitário Cassiano Antonio Moraes – UFES.

**Table 1:** Recruitment facilities, services provided, and collaborating research institute

	City/State	Clinic name	Collaborating Research Institute	Clinic type	Population
1	Fortaleza/Ceará	Centro de Saúde Carlos Ribeiro	Universidade de Fortaleza	Public STI/HIV	Patients undergoing HIV/STI screening/testing
2	Fortaleza/Ceará	Centro de Saúde Meireles	Universidade de Fortaleza	Public STI/HIV	Patients presenting for STI symptoms
3	Fortaleza/Ceará	Universidade Federal	Universidade de Fortaleza	Specialty STI referral	STI symptoms and referrals for diagnosis and treatment
4	Pelotas/Rio Grande do Sul	Ambulatório de Ginecologia e Obstetrícia - Faculdade de Medicina.	Universidade Federal de Pelotas	ObGyn Universidade Federal de Pelotas	Female patients For ObGyn evaluation
5	Pelotas/Rio Grande do Sul	Ambulatório de Ginecologia e Obstetrícia - Campus da Saúde	Universidade Federal de Pelotas	ObGyn Universidade Católica de Pelotas	Female patients For ObGyn evaluation
6	Vitória/Espírito Santo	Hospital Universitário Cassiano Antonio Moraes - UFES	Departamento de Medicina Universidade Federal do Espírito Santo	University Hospital	ObGyn and Infectious diseases outpatients clinics

The three clinics in Fortaleza are directed by the state and municipal health authorities. These clinics have a research agreement with UNIFOR. The clinic in Vitória is directed by the local health authority and has a research agreement with the UFES. The two clinics in Pelotas are directed by and have a research agreement with the Federal University of Pelotas. The public universities in Vitória and Pelotas fall under the research direction of the Brazil Ministry of Health. UNIFOR is a foundation that is not under the direction of the Brazil Ministry of Health. The laboratories in Fortaleza and Vitoria are public health laboratories. The laboratory in Pelotas is a private laboratory (Table 2).

**Table 2:** Laboratories that will provide syphilis RPR testing

City/State	Laboratory name	Location	Collaborating Research Institute
Fortaleza/Ceará	Laboratorio Central - LACEN	Av, Barao de Studart, 2405 – Dioniso Torres, Fortaleza	Universidade de Fortaleza
Pelotas/Rio Grande do Sul	Laboratorio Antonello	R. Padre Anchieta, 1620 – Centro, Pelotas	Universidade Federal de Pelotas
Vitória/Espírito Santo	University Hospital laboratory	Av. Mal. Campos, 1355, Vitória - ES, 29043–260	Universidade Federal do Espírito Santo

The facilities and services provided at each clinic are described in Table 1. Documents required for the national ethics review of research studies conducted in Brazil that document access to the clinic and patient records are available upon request. Each of the participating clinics and attending staff has the experience and capacity to work with the investigators to implement the protocol.

A distribution of non-treponemal titers among pregnant women diagnosed with syphilis demonstrates high numbers of women with non-treponemal RPR titers ≥1:16 diagnosed in various clinics in Fortaleza, Vitoria and Pelotas. The timing of the recruitment process will depend on the number of women diagnosed with syphilis and who have an RPR titer ≥1:16 and who consent to participate in the study.

### Participant recruitment

Patients identified during standard of care testing with a positive rapid syphilis test will be screened for eligibility and consented for testing with RPR used for the study (Becton Dickinson, U.S.A.) and for participation in the study. Those with a titer of ≥1:16 will be offered enrollment according to the following description.

**Fig. 1:**
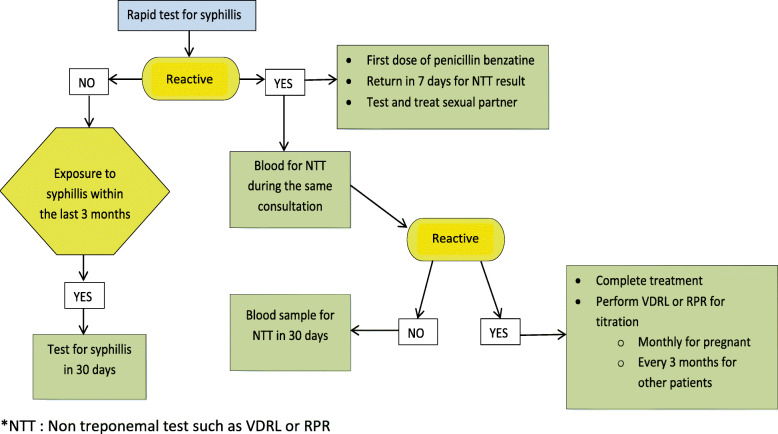
Standard care for syphilis diagnosis and treatment as per the Brazil Ministry of Health guidelines [34]. *NTT: Non treponemal test such as VDRL or RPR

Non-study clinic-site staff will perform the first screening rapid finger-stick test for syphilis. The rapid syphilis test currently used in these clinics detects both new (acute) infection and old treated infection but does not distinguish between the two. Patients that are positive for the syphilis rapid finger-stick test and who meet other criteria for eligibility will be referred to a study nurse to obtain consent. Following consent, blood will be drawn by a study nurse for RPR testing. HIV and pregnancy testing will be performed. Patients will be notified of RPR results in approximately three days to one week by a study nurse. Patients with RPR test titers ≥1:16, HIV negative and non-pregnant will be randomized into one of the study groups. Those with RPR titers < 1:16 will receive standard of care treatment for syphilis according to national guidelines (Fig. 1) [34]. It is expected that an enrolment visit could last approximately 1.5 hour.

Patients that are not eligible for the study (negative RPR or RPR with titer < 1:16) will receive syphilis RPR results and treatment according to national syphilis treatment recommendations from a study nurse. Pregnancy tests and HIV tests will be completed on all potential participants and those that test positive will not be enrolled in the study.

To benchmark the performance of BPG in the study population randomized to receive cefixime, a contemporary arm of participants that will receive standard of care treatment with BPG according to the Brazil national STI treatment guidelines will be included. A ratio of two patients receiving cefixime to one patient receiving BPG will be used.

This non-comparator arm of BPG will be included to account for study population differences in terms of stage of disease, history of prior infection, experience of re-infection, experience of serofast state, or other co-factors that could impact serological response. An estimate of performance of this standard of care will be calculated, but this will not be compared directly to the study intervention as the study is not powered to assess statistical significance between these groups.

### Randomization

We will enroll 210 women into the study: 140 for the cefixime arm and 70 for the BPG arm in a randomly allocated ratio of 2:1.

The Statistical Analysis System (SAS) Version 9.1 program will be used for generating a randomization sequence. The SAS-generated randomization sequence will be stratified by centre, in randomly permuted blocks of sizes 3, 6 and 9. The randomization list and allocation sequence will be developed by the WHO statistics team and will be distributed to the clinics by the study team. Information regarding number of blocks and block sizes will only be available to the WHO trial statistician. An example of the randomization generation is presented in Additional file 1.

Sealed envelopes containing group assignment will be prepared at the study coordinating centre (WHO) and will be marked externally only with the randomization sequence number. The assigned randomization envelope will be opened once all enrolment procedures are complete. Study nurses will enroll participants and will assign participants to interventions. This is an unblinded trial. Participants and study staff will be aware of the intervention assignment.

Patients will be participating in the study for approximately nine months. The study is expected to last for approximately two years. Women that are referred to the study but decline enrolment will be treated at the clinic following national STI treatment guidelines for syphilis.

***Inclusion Criteria***:

1. Female, 18 years of age or older
2. Non-pregnant
3. Able to provide informed consent
4. Test positive for syphilis with a positive rapid treponemal test **and** an RPR titer equal to or greater than 1:16
5. Non-cephalosporin allergic
6. Non-penicillin allergic
7. Agree to be called once-a-day by study staff to be reminded to take study drug
8. Able to swallow pills
9. Willing to attend follow-up visits at 14 days, 3, 6, and 9 months after completion of the study treatment
10. Willing to take oral contraceptive or use condom to prevent pregnancy during the study period
11. HIV negative

***Exclusion Criteria*** for the intervention include:

1. Female under 18 years of age
2. HIV positive
3. Pregnancy test positive or clinical pregnancy
4. Breastfeeding*
5. Prior history of syphilis or syphilis treatment**
6. Allergy or contraindication to penicillin or cephalosporins (including allergy to cefixime)
7. In the judgment of the interviewer, has a medical condition or other factor that might affect her ability to follow the protocol
8. Previous enrollment in the study
9. Presenting a situation or condition that would not allow reliable study follow up (For example: frequent travel, visitor to area, alcohol abuse or substance misuse)
10. Lacking mental capacity to give informed consent to participation
  *Breastfeeding was added as an exclusion criterion as an amendment after enrolment initiation on January 24, 2020.
  **History of receiving any antibiotic within the previous six months prior to screening for eligibility was removed as an exclusion criterion as a protocol amendment after enrolment initiation on January 24, 2020.

### Schedule and content of study follow up visits and telephone encounters

#### Study Visit 1 at day zero: Non-pregnant, HIV negative women with positive treponemal test and RPR titer ≥1:16 and meeting inclusion criteria will be offered enrolment

Informed consent will be completed by a study nurse prior to RPR testing, and baseline clinical information including symptom review, medical and sexual history, physical exam will be collected. Patients not consenting to participate in the study will receive treatment per national treatment guidelines for syphilis.

Subject data will be collected on study specific case report forms. Baseline treponemal result and RPR titer, signs and/or symptoms of syphilis and other STIs, will also be collected. Data will be entered into a central database using patient identification numbers only. Venipuncture will be performed, and blood will be collected for RPR quantitative titers Table 3.

**Table 3:** Schedule of venipuncture specimen collection visits

Visit number	Scheduled visit	Allowed specimen collection interval outside of scheduled visit date
1	Screening	RPR test, HIV (eligibility checking)
2	Enrolment	Not applicable
3	3 months	10 days before or 45 days after
4	6 months	44 days before or 45 days after
5	9 months	44 days before or 45 days after

Once specimens have been collected and RPR results return at values equal to or greater than 1:16, subjects will be randomly assigned to one of two groups:

- Group A: standard of care or penicillin group
- Group B: experimental group or cefixime group

Participants of Group A will receive treatment for syphilis with BPG according to national STI treatment guidelines. Participants in group A will be instructed to remain in the clinic for observation for 30 minutes after receiving this penicillin injection. According to national syphilis treatment guidelines, patients receiving penicillin may be asked to return for additional injections. These study participants will be asked to visit the clinic 3, 6 and 9 months after the day they received BPG for evaluation and RPR testing. BPG will be provided by the Brazil Ministry of Health.

Participants of Group B will receive their first dose of 400 mg cefixime at the study clinic under direct observation. Patients receiving cefixime will be instructed to remain in the clinic for observation for 30 minutes after receiving the medication. Participants will be given the cefixime pills and instructed on how and when to take their medication at home which they must continue for 10 days. Daily phone calls and text messages will be made to the patients by a study nurse to ascertain adherence to study medication and record side effects. Patients will be asked to bring back any unused study medication to their next visit two weeks following enrolment. Study participants receiving cefixime will also be asked to return to the clinic for scheduled visits 3, 6 and 9 months after the day of the last dose of cefixime for evaluation and RPR testing.

#### Study Visit 2 at two weeks following enrolment (allowable two days before or five days after scheduled visit):

Participants receiving cefixime will return to the clinic two weeks after enrollment for assessment of treatment compliance, evaluation resolution of symptoms if present at enrollment, and to ascertain any toxicities. Subjects will be asked to return for a scheduled clinical and laboratory assessment (Study Visit 3) at three months following enrollment.

### Phone call at 30 days following enrolment

A phone call to study participants randomized to cefixime will be conducted to perform a final evaluation of safety (side effects and adverse events) following the completion of the course of study drug.

#### Study Visit 3 at three months following enrolment (allowable 10 days before or 45 days after scheduled visit):

At Study Visit 3, subjects that received either cefixime or BPG will be asked questions regarding their current symptoms, interval sexual history, concomitant antibiotic use and possible adverse reactions. Subjects will have a venipuncture blood specimen collected for syphilis testing (RPR). For clinical safety, those subjects who experience a fourfold (two titer) increase in RPR titer at three months post treatment will be considered a treatment failure and will receive benzathine penicillin treatment according to national guidelines. All participants will undergo pregnancy testing. Those that are found positive on pregnancy testing will be treated for syphilis according to national guidelines and removed from the study. Subjects that received either cefixime or BPG will be asked to return at six months from the time of treatment.

#### Study Visit 4 at six months following enrolment (allowable 44 days before or 45 days after scheduled visit):

Subjects that received either cefixime or BPG will be asked questions regarding their current symptoms, interval sexual history, concomitant antibiotic use and possible adverse reactions if they received cefixime. Subjects will have a venipuncture blood specimen collected for syphilis testing (RPR) increase in RPR titer at 3 or 6 months post treatment. Those subjects who experience a fourfold (two titer) increase in RPR titer at three or six months post treatment will be considered a treatment failure and will receive BPG 2.4 million units IM once per week three weeks. Data for these patients through Study Visit 4 will be included in analyses, and for analysis purposes, these patients will be considered to be treatment failures. All participants will undergo pregnancy testing. Those that are found positive on pregnancy testing will be treated for syphilis according to national guidelines and removed from the study. A voluntary repeat HIV test will be performed at 6 months. Patients that test positive for HIV at 6 months will be referred for HIV care. No additional treatment for syphilis will be provided based on HIV-positive status other than according to the study protocol. Response to treatment of these patients will be included in the analysis. Subjects that received either cefixime or BPG will be asked to return at 9 months from the time of treatment.

#### Study Visit 5 at 9 months following enrolment (allowable 44 days before or 45 days after scheduled visit):

Subjects that received either cefixime or BPG will be asked questions regarding their current symptoms, interval sexual history, concomitant antibiotic use and possible adverse reactions. Subjects will have a venipuncture blood specimen collected for syphilis testing (RPR). All participants will undergo pregnancy testing. Those that are found positive on pregnancy testing will be treated for syphilis according to national guidelines.

**Note:** A repeat venipuncture for serum collection at specimen collection follow-up visits (enrolment, 3, 6, and 9 months) will be performed if the initial specimen is determined to be inadequate following on-site centrifuge. Centrifuge and evaluation of specimen adequacy will be performed within 30 min of specimen collection. It is expected that repeat collection will rarely be needed as trained venipuncture staff will be responsible for collecting specimens. However, hemolysis can occur regardless of the venipuncture technique and this can contaminate the RPR assay and results.

**Reimbursement to subjects:** Study subjects, and their sexual partners who are referred to the study clinics for evaluation and treatment, will be reimbursed for their time and effort to participate in the study per local ethical authority guidelines.

**Partner referral and treatment:** All subjects will be encouraged to refer all partners with whom they have had vaginal, oral, or anal sex in the past 60 days to the clinic for routine evaluation and treatment. Upon presentation to the clinic, all partners will be tested and if positive, offered treatment according to local clinic guidelines.

### Discontinuation of study enrolment

1. Patients found to be pregnant at follow up visits will be removed from the study and given treatment with BPG based on stage and national guidelines. Patients that originally received one penicillin injection will receive treatment based on national guidelines.
2. Women reporting a toxicity of grade 3 or greater, that are definitely or probably related to the study drug cefixime (to be determined by a physician not associated with the study), during the treatment period will be removed from the study.

### Main variables

Variables collected at time of enrolment will include age, past history of syphilis, RPR titer and HIV status.

The primary variables for outcome analysis will include:

1. The change in RPR titer between the baseline visit and by six months post enrolment.
2. Safety and tolerability of study drug.

### Outcome measures

#### Primary outcome measure

The primary study outcome will be based on the change in the RPR titer from baseline to six months. Those subjects who have a 4-fold decrease in RPR titers from baseline by six months will be considered a positive treatment response (treatment success). All other participant outcomes will be defined as treatment failure including those subjects who experienced a fourfold (two titer) increase in RPR titer at three or six months post treatment and received BPG 2.4 million units IM once per week for 3 weeks.

**Note:** Subjects that received either cefixime or BPG that experience a 4-fold titer decline by three or months followed by a 4-fold titer rise by six or nine months will be classified as (1) treatment success at three months and (2) syphilis re-infection during the period following the initial 4-fold RPR titer decline.

**Note:** While there are limited data on the use of cefixime for the treatment of syphilis, cefixime is a broad spectrum, third generation cephalosporin in the same class and family as ceftriaxone for which extensive data on treatment efficacy for syphilis are available. RPR titers will be monitored frequently in this study and treatment with BPG will be provided if RPR titer increases fourfold (2 titer) at 3 or 6 months or does not reflect an adequate response (fourfold or 2-titer decline) at 6 months.

#### Secondary outcome measure

The secondary outcome measure is the safety and tolerability of cefixime as a treatment for syphilis.

This will be measured by the percent of treated patients experiencing mild, moderate, severe, or life-threatening adverse events, either temporally associated or not associated with the study product. Patients will be instructed on the possible side effects of the study drug, and when to call the study team should they experience any of these side effects. We will use the Common Terminology Criteria for Adverse Events (CTCAE) [35] to evaluate side effects and the severity of grade (scale 1–4). Women reporting a toxicity of grade 3 or greater, that is or probably related to study drug (to be determined by a physician not associated with the study), during the treatment period will be asked to stop study treatment, return to the clinic, and they will be treated with 2.4 million units of BPG. Patients will be followed closely until the toxicity returns to a grade 2 or less.

### Sample size considerations

The primary analysis will compute the proportion of subjects with a 4-fold decrease (from study entry RPR) in RPR titers from baseline by six months in the per-protocol (PP) analysis population.

We have powered this study to detect a significant difference from a minimal accepted efficacy of standard treatment with benzathine penicillin (80%). This minimal efficacy was selected based on a multi-center clinical trial evaluating the efficacy of penicillin versus azithromycin [36].

A 90% response rate for cefixime was selected based on recent meta-analyses, a multi-center trial of ceftriaxone for treatment of syphilis [10, 11, 37, 38]. To calculate the number of subjects required to enroll to reach 94 evaluable subjects in the PP analysis population, the following assumptions are made:

Using a two-sided exact binomial test at α = 5%, a sample size of 107 achieves an 80% power to detect a minimum acceptable cefixime efficacy (p_0_) of 0.80, assuming the anticipated efficacy proportion (p) of 0.90. Assuming 20% dropout rate and other related factors, the final sample size for the cefixime arm is inflated to *N* = 140, with correspondingly 70 participants recruited in the BPG arm, in a ratio (cefixime to BPG) of 2:1 (Table 4).

**Table 4:** Sample size to detect a minimum acceptable efficacy (p_0_) of 0.80 using a two-sided Exact binomial test for difference assuming alpha = 5% and power = 80%*

Anticipated efficacy *P*	Sample size *	Effective sample size (20% inflated) *n*
0.86	316	395
0.87	225	282
0.88	173	217
0.89	131	164
0.90	107	**134**
0.91	88	110
0.92	69	87
0.93	55	69
0.94	48	60
0.95	41	52
0.96	34	43
0.97	26	33
0.98	26	33
0.99	17	22

### Analysis populations

#### Per-protocol (PP) population

This analysis population includes enrolled subjects who met all inclusion/exclusion criteria, complied with study treatment, and had RPR test results at the 6-month visit. To be compliant with study treatment, a subject must be able to swallow the pills and not vomit within 30 minutes of administration. No more than two consecutive missed doses will be considered as compliant with study treatment. Completion of at least 7 days of treatment or at least 14 capsules of 400 mg of cefixime will be considered as study compliant.

#### Intent-to-treat (ITT) population

This analysis population includes all enrolled subjects. We will conduct daily phone calls to the study participants to ensure adherence to study medications. Study participants will receive reminder texts to take their medication for the evening dose of medication. We will give reminder calls to patients prior to their scheduled follow up visits.

### Baseline characteristics

Baseline and demographic characteristics will be summarized. For both continuous and categorical variables, appropriate summary statistics will be applied. For continuous variables, descriptive statistics will include the number of non-missing values, mean, and standard deviation, median, minimum, and maximum. For categorical variables, descriptive statistics will include counts and percentages per category.

### Efficacy and other analysis plan

#### Study population and follow-up status

The number of participants screened, eligible, randomized and with follow-up status (lost-to-follow-up, withdrawn or completed study follow-up) will be tracked, as well as the numbers analyzed in the per-protocol or by intention-to-treat population, by allocated arm.

- Baseline characteristics of the study population will be summarized by allocated arm
- Follow-up status at 3, 6 and 9 months showing the number of participants who came back for the visit will be tabulated by allocation arm
- Tabulated summary showing treatment adherence to cefixime and description of those who received the rescue therapy with BPG will be presented. Treatment adherence to cefixime is defined as patients taking all doses of cefixime or at least 14 doses without more than two consecutive missed doses.

### Study outcomes evaluation

#### Primary analysis

The primary outcome is the proportion cured using cefixime treatment, evaluated at six months post-recruitment.

Proportion representing proportion cured using cefixime treatment success, cure defined as at least 4-fold reduction in RPR titer by six months (compared to baseline). Participants experience a 4-fold reduction in RPR before six months will be counted as a success at six months.

The primary analysis will assess whether the proportion cured is greater than or equal to 90% and whether the observed efficacy is statistically different than the minimally acceptable efficacy set at 80%. Proportion cured and 95% confidence intervals will be calculated using the binomial exact test. Cefixime will be declared efficacious if the lower limit for a two-sided 95% confidence interval (CI)≥ 0.80.

- *Cure:* At least a 4-fold decrease in RPR from baseline to 6 months.
- *Failure:* Less than a 4-fold decrease in RPR titer from baseline to 6 months, or an increase in RPR titer.
- *Non-evaluable:* At least one of the following: Subject did not return for 6 month visit, or RPR results from the 6 month visit were not available for any reason, or subject could not be evaluated for any other reason.

For the PP analysis population, subjects with non-evaluable outcome status will be excluded from the analysis. For the ITT analysis population, subjects with non-evaluable outcome status will be included in analyses and classified as treatment failures. Subjects who discontinued early from the study due to a lack of treatment effect are included in all analysis populations as treatment failures.

The primary efficacy analysis will be based on the cure rate by 6 months and will be performed in the PP analysis population. The primary analysis will also be performed in the ITT population, but this is a secondary outcome measure.

#### Secondary analysis

Secondary outcome measures will include the safety and tolerability of the study drug. These end points will be assessed among all subjects who have taken at least one dose of trial medication (including subjects who vomit within 1 hour of study drug administration).

The number of safety events* as measured by side effects and adverse events as well as tolerability reported by participants will be evaluated. The safety events will be tabulated by treatment allocation, event grade, severity and relatedness to study treatment [35]. The tolerability will be defined as the ability of participants to ingest all or at least 7-days or 14 doses of cefixime without experiencing treatment-related side effects or adverse events that cause the patient to discontinue the medication or that prevent the participant’s ability to complete the full course of treatment. The number of participants discontinuing therapy due to intolerance of cefixime will be reported.

*The types of safety events as collected on the side effects and adverse reporting questionnaires will be based on the period of medication administration (10 days) and at 14 and 30 days following the first dose of treatment.

### Data collection and management

Study data will be collected on paper case report forms (CRFs) approved by the internal form review committee and key-entered at the respective collaborating research institute. The WHO study team in Geneva has developed a customized web-based electronic data capture (EDC) system using Open Clinica Enterprise platform for entering, cleaning, and tracking study data. The web-based data capture system will be designed to ensure that only authorized staff can enter, change, or view data. The local study data management teams will be trained in data collection, online data entry, and data management using the EDC system. The EDC system will perform edit checks during the data entry process to notify immediately of potential errors and inconsistencies. Using this system, the data will be available in the study database and accessible to the WHO study team for review of information as soon as the data are entered at the local site. Double data entry will be used. The local study team will keep an updated log of screened and enrolled study participants. WHO team will liaise with the local study team to coordinate and track CRF completion and data queries. The database will be locked after all queries have been resolved and analyzed at WHO.

### Data safety and study monitoring

The study will be followed by a Data Safety Monitoring Board (DSMB). The DSMB will be composed of 5 members: (1) Physician/Clinical researcher; (2) Biostatistician; (3) Ethicist; (4) Epidemiologist; and (5) Infectious diseases, microbiologist. A data safety and monitoring plan (DSMP) includes the terms of reference for the DSMB, interim analysis tables and study stopping rules. This DSMP is available upon request (mtaylor@who.int; karae@who.int).

External monitoring of clinic recruitment sites will be performed by Fiocruz, Brazil at study initiation, during three follow up visits and at study completion.

The data monitoring committee consists of 2 WHO biostatisticians, one clinical trials data collection and monitoring expert, two WHO medical officers responsible for the trial and one non-study related WHO staff.

### Ethical concerns

#### Potential risks

1. Potential risks to study participants include the possibility that the alternative treatment may not prove to be effective. For this reason, we will closely follow the subjects for adherence to study medication including daily phone or in-person contact to establish adherence to study drug. RPR titers will be collected 12 weeks after completion of treatment (with no more than 5-day result turnaround time). If the participant experiences an RPR titer increase of 4- fold or more from the entry RPR at 3 or 6 months, we will consider this a treatment failure and the participant and be given 2.4 million units of BPG immediately and according to national guidelines.
2. Risks to privacy: Participants’ attendance at study visits may be accidentally noticed by others.
3. Psychological risks: Receiving a positive syphilis diagnosis could be upsetting to a participant. However, syphilis diagnosis occurs as part of standard clinical care and outside of the study.
4. *Risks associated with blood draw*: Participants may experience discomfort, bruising, or even fainting in connection with the blood draw that will be performed during the visits. There is also a slight risk of infection.
5. Cefixime is contraindicated in patients with known allergy to cefixime or other cephalosporins. Some participants may not be aware of their allergy status. However, anaphylactic or anaphylactoid reactions (including shock and fatalities) have rarely been reported with the use of cefixime, thus this risk is considered to be very low.
6. Some adverse reactions were documented in other cefixime clinical trials. In United States (U.S.) trials of the tablet formulation of cefixime, the most commonly seen adverse reactions were gastrointestinal events. These events were reported among 30% of adult patients that received either the twice daily or the once daily regimen. Discontinuation of cefixime during the U.S. clinical trials occurred among 5% of study participants because of drug-related adverse reactions. Other individual adverse reactions reported from U.S. clinical trials included diarrhea (16%), nausea (7%), loose or frequent stools (6%), flatulence (4%), abdominal pain (3%), and dyspepsia (3%). Observed and reported adverse reactions for cephalosporins including but not limited to cefixime have included allergic reactions, superinfection, renal dysfunction, toxic nephropathy, hepatic dysfunction including cholestasis, aplastic anemia, hemolytic anemia, hemorrhage, and colitis.
7. In this study, participants will be observed for 30 minutes in the clinic following the first dose of cefixime. In the event of an adverse event or an allergic reaction possibly related to cefixime, participants will be evaluated by a study nurse and by a clinic-based study physician. Participants will be referred to emergency services at the discretion of the clinic-based study physician. Cefixime will be discontinued in the event of an allergic reaction.
8. The efficacy of cefixime to prevent mother-to-child transmission of syphilis is unknown. Study participants will be counseled regarding the risk of untreated syphilis in pregnancy. Each participant will be offered oral contraceptive tablets as well as condoms to prevent pregnancy for the duration of the study. Condoms will be provided to each participant and use will be encouraged at each visit to reduce the risk of re-infection with syphilis and infection with other STIs.

### Risk management procedures and adequacy of resources

1. Blood draw: We will ensure that the staff person performing the blood draw is a trained and certified phlebotomist. All standard precautions against infection will be taken.
2. Before therapy with cefixime is instituted, careful inquiry will be made to determine whether the patient has had previous hypersensitivity reactions to cephalosporins, penicillins, or other drugs.
3. Patients found to be pregnant at follow-up visits will be removed from the study and given an injection of 2.4 million units of BPG.
4. Sexual partners of enrolled patients will be solicited and offered treatment as per the national guidelines.
5. Patients will be offered contraception for the duration of the study to prevent pregnancy that could be affected by untreated or under-treated syphilis. Oral or injectable contraceptive will be offered to patients according the choices available on clinic-site formularies.

If a study participant suffers an injury or illness related to this research, they will be instructed to contact the Principal Investigator immediately. If it is determined by a doctor, not associated with this study, that the injury or illness is definitely related to the study drug “cefixime” or a study related procedure, medical care will be provided or arranged. Costs associated with study-related injuries (physical, psychological, social harm, or loss of wages) will be covered by the study at no cost to the participant. In no way does signing the consent form waive their legal rights nor does it relieve the investigators or involved institutions from their legal and professional responsibilities. This information is included in the consent form.

### Privacy and confidentiality considerations including data access and management

#### Consenting, screening, and study visit

The informed consent procedure is designed to maximize the potential participant’s comprehension of study procedures and to ensure that participation is voluntary. Before a participant is enrolled, a study nurse will explain the study’s purpose, the procedures to be followed, and the risks and benefits of participation. A copy of the consent form, which includes a description of the study, will be provided to all participants. The study nurse will assess whether the potential participant has understood the study and consent form by asking key questions related to participation. If a potential participant decides at any point during the informed consent process that they do not wish to participate, her decision will be honored regardless of how well she comprehends the study information.

The consent process will be conducted in a private exam room. The level of linguistic difficulty (vocabulary, sentence length and complexity, etc.) will be kept at a basic (approximately fourth-grade) level to ensure maximum comprehension. Once written informed consent is obtained, the first study procedure (specimen collection) will take place immediately.

#### Confidentiality of participant data

The first study data collection form will include the Patient Identification Number (PID) and the associated participant’s personal information in the event of a need to contact the participant during the study. This form will be kept in a separate locked file and will not be included in the study record. Study forms that will remain in the study file will only contain the patient PID.

All additional data collected from enrolled participants, including both hard copy and electronic data and biological samples, will be identified only by the participant’s study ID and will be physically protected against access by anyone except authorized staff connected to the study. Hard copy data will be stored in locked cabinets in secure offices, while electronic data will be password-protected. The computer file that contains the key to participants’ code numbers (name-to-ID relational file) will be encrypted, and the computers on which it resides will be locked in each site manager’s office. Original signed consent forms will be stored in a locked file cabinet as well as participant’s contact information.

The use of the national laboratory information system called Gerenciador de Ambiente Laboratorial (GAL) will be programmed for entry of the laboratory results of enrolled using study ID numbers in a restricted, study-specific portal per standard national research methods. Results will be available only to the site-specific study staff accessible only by participant study ID number.

#### Banking of samples

Participants will be informed in the consent document of the storage of their samples until the end of the study. Blood samples collected during this study will be stored only until the end of the study. At the end of the study all samples will be destroyed. Each of the research facilities has completed a bio-banking agreement. Samples collected during this study will be entrusted to the designated university-level research entity (Table 1) and stored at the related laboratory facilities (Table 2).

#### Data sharing

The Principal Investigators (PIs) will share de-identified participant data with other study investigators and co-principal investigators at other study sites.

#### Communication of results

The sponsor and investigators will communicate trial results to participants, healthcare professionals, the public, and other relevant groups via publication, reporting in results databases, and other data sharing arrangements following institutional publication clearances and approvals.

#### Laboratory quality assurance

A subsample of stored aliquots of sera/plasma from enrolled participants will be shipped to the Brazil National Reference Laboratory to perform repeat laboratory-based treponemal and non-treponemal qualitative and quantitative testing for this study. This laboratory participates in the WHO/U.S. CDC syphilis quality assurance program. This will be coordinated by Professora Maria Luiza Bazzo, Laboratório de Biologia Molecular, Microbiologia e Sorologia – LBMMS, Centro de Ciências da Saúde / UFSC.

#### Compensation for participation

Study medication will be provided by the study at no charge to patients. Participants and their sexual partner (or accompanying person when necessary) will receive reimbursement, at each visit to the study clinics, that is determined as locally adequate for their time and transportation expenses without being coercive.

#### Research capacity strengthening

Because of the importance of this study we are selecting study sites in Brazil with experience in conducting clinical trials.

#### Project organization

The organization follows recruitment, treatment and follow up of 210 patients diagnosed with infectious syphilis (RPR titer 1:16 or greater) at 6 clinics in the three cities of Fortaleza, Vitória and Pelotas, Brazil. Dr. Melanie Taylor (WHO), Dr. Nathalie Broutet (WHO), and Dr. Edna Kara (WHO) will assist the principal investigators Dr. Alix Araujo, Dr. Angelica Miranda, Dr. Mariangela Silveira, Dr. Ivo Castelo Branco and site coordinators to oversee protocol implementation and patient follow-up at the study clinics in Brazil. Protocol visit implementation and study monitoring visits will be performed by WHO study staff and external monitoring professionals at pre-established schedules.

## Discussion

This trial holds potential benefits and significance to the sexual and reproductive health and rights research area through the identification of an alternative treatment for syphilis. The antibiotic, cefixime, used in this study has received FDA marketing approval for use in the U.S. for at least one clinical indication by the FDA, and has been in use for greater than 10 years [5]. The wholesale cost of cefixime 400 mg is US $26.60/100 tabs (IDA pharmaceuticals, Netherlands, Nectar Lifesciences, India). This is an approximate cost of $5.32 per study regimen. This study may provide an alternative regimen for the treatment of syphilis in cases of penicillin allergy or unavailability of BPG. It may also provide an oral regimen for settings in which injections are not feasible. Results demonstrating efficacy of cefixime for the treatment from this Phase II trial can inform a future proposed randomized controlled trial of cefixime treatment for pregnant women with syphilis and prevention of congenital syphilis in support of global goals of elimination of mother-to-child transmission of syphilis [39–41]. It is not expected or intended that this regimen would replace the primary recommendation of low-cost BPG as first line treatment for syphilis in any population group.

## Supplementary information

**Additional file 1.** Randomization of participants following the permuted block randomization 2:1 method.

## Data Availability

Not applicable. Data are not available due to ongoing collection.

## Abbreviations

RPR: Rapid plasma reagin
IM: Intramuscular
CRF: Case report form
EDC: Electronic data capture
MIC: Minimum inhibitory concentration
CTCAE: Common Terminology Criteria for Adverse Events
DSMB: Data Safety Monitoring Board
UNIFOR: Universidade de Fortaleza
STI: Sexually transmitted infection
WHO: World Health Organization
WHOERC: World Health Organization Ethics Review Committee
CONEP: Comissão Nacional de Ética em Pesquisa
UFES: Centro de Ciências da Saúde da Universidade Federal do Espirito Santo
UCPel: Universidade Catolica de Pelotas
UFPel: Faculdade de Medicina da Universidade Federal de Pelotas
UFC: Universidade Federal do Ceara
CDC: U.S. Centers for Disease Control and Prevention, National Center for HIV, Hepatitis, STD, and TB Prevention
ITT: Intent to Treat Analysis
IV: Intravenous
MBC: minimum bactericidal concentration
NTT: Non treponemal test
PID: Patient identification
PI: Principal investigator
US: United States
FDA: United States Food and Drug Administration
VDRL: Venereal Disease Research Lab
